# 

**Published:** 1870-04

**Authors:** 


### OUR EXCHANGES.

THE LYCEUM BANNER.—This is the title of a semi-monthly journal for young people, published at Chicago, Illinois, by Low H. Kimball, 137½ Madison Street. It is really an excellent little magazine—just such an one as you would like to have your little ones read. The price is only $1.00 a year. Send for a specimen copy.

THE UNIVERSE has been greatly improved since its removal from Chicago to New York. We regard it as the most entertaining and spicy journal that comes to our sanctum. If you want a large dose of common sense for $3.00 a year, subscribe for the *Universe*, by sending money and address to H. N. F. Lewis, corner Broadway and 32d Street, New York.

THE EUROPEAN MAIL.—If you desire to learn of everything that is transpiring across the water, to know of the whereabouts of Americans in Europe; and if you wish the reports of stocks, the markets, shipping, mercantile affairs and literary matters, subscribe for the above named journal. Address:—*The European Mail*, London, England.

THE TECHNOLOGIST is the title of a very handsome magazine, published by "The Industrial Pub. Co.," at 176 Broadway, N. Y, at $2.00 a year. It is devoted especially to Engineering, Manufacturing and Building, is beautifully printed and illustrated, and is ably edited by a corps of well-known, scientific men. The designs for building are alone worth the price of subscription, to say nothing of the valuable articles it contains upon all manner of industrial pursuits.

ZELL'S ENCYCLOPEDIA, published by T. Elwood Zell, at Philadelphia, is meeting with a large sale throughout the country, as it deserves to. It is by far the best Universal Dictionary ever published.

